# Suppression of X-ray-induced dissociation of H_2_O molecules in dense ice under pressure

**DOI:** 10.1038/srep26641

**Published:** 2016-05-25

**Authors:** Hiroshi Fukui, Nozomu Hiraoka, Naohisa Hirao, Katsutoshi Aoki, Yuichi Akahama

**Affiliations:** 1 Center for Novel Material Science under Multi-Extreme Conditions, Graduate School of Material Science, University of Hyogo, 3-2-1 Kouto, Kamigori, Hyogo 678-1297, Japan; 2 SPring-8 Taiwan Beamline Office, National Synchrotron Radiation Research Center, Hsinchu 30076, Taiwan; 3 Japan Synchrotron Radiation Research Institute, 1-1-1 Kouto, Sayo, Hyogo 679-5198, Japan; 4 Geochemical Research Center, Graduate School of Science, The University of Tokyo, 7-3-1 Hongo, Bunkyo-ku, Tokyo 113-0033, Japan

## Abstract

We investigated molecular dissociation induced by 10-keV X-ray irradiation in dense ice at pressures up to 40 GPa at 300 K. The dissociation yield estimated from the oxygen *K*-edge X-ray Raman spectra, showed that the molecular dissociation was enhanced up to 14 GPa and gradually suppressed on further compression to 40 GPa. The molecular dissociation was detected for a rather narrow pressure span of 2–40 GPa by the X-ray spectroscopy. The pressure variation of the dissociation yield was similar to that observed in the electric conductivity of ice VII and likely interpreted in terms of proton mobility.

Water ice exhibits various thermodynamically stable and quasi-stable phases at temperatures up to 2000 K and pressures up to 100 GPa^1^. Each phase has a characteristic network structure of water molecules, depending on pressure and temperature conditions. At high pressures above 2 GPa, ice has high-density packing structures consisting of interpenetrating diamond-like sublattices, in which each oxygen atom is surrounded by eight nearest neighbors but is connected to only tetrahedrally coordinated four neighbors with hydrogen bonds. Ices with a body-centered sublattice of oxygen atoms are called dense ices; they are ice VII, ice VIII, and ice X.

Dense ice has attracted broad interest in the fields of physics, chemistry, materials science, and earth and planetary sciences. There are some studies reporting the hydrogen bonding, hydrogen sublattice, or hydrogen mobility in dense ice significantly change on compression even though the body-centered arrangement of oxygen atoms remains unchanged. Careful analyses of the compressional equation of state of dense ice^2^,^3^ revealed that an intermediate state exists between ices VII and X at room temperature. A Raman spectroscopy study indicated that there is a phase boundary in the stability region of ice VII (around 14 GPa and at temperatures below 100 K)^4^. X-ray and neutron diffraction studies at 300 K also revealed anomalies in diffraction intensity around this pressure^5^,^6^. Although the details of the anomalies are still unknown, some changes in the hydrogen behavior in the oxygen sublattice seem to be the origins of these anomalies in dense ice because the body centered oxygen sublattie is unchanged. A study of electric conductivity of dense ice showed the maximum at the similar pressure condition and discussed that the major charge carriers changed from the rotational defects to the ionic defects with compression^7^.

Another phenomenon suggesting “instability” of the hydrogen bonds in dense ice was observed at pressures up to 15 GPa: Ice VII undergoes photochemical reaction or dissociation of H_2_O molecules by irradiation of intense 10-keV X-ray^8^. This study argued that a dissociation product was an alloy of H_2_ and O_2_ molecules^8^. A follow-up study by optical laser Raman spectroscopy (ORS)^9^ revealed two events related to the dissociation product: O_2_ molecules produced at 17.6–22.5 GPa persisted stably for two years under the confined pressure or on further compression up to 70 GPa.

We investigated the electronic structure of oxygen, the molecular vibrations, and the atomistic structure for X-ray-irradiated dense ice by means of X-ray Raman scattering (XRS), ORS, and X-ray diffraction (XRD), respectively, and revealed that the molecular dissociation was suppressed at pressures above 40 GPa. The X-ray-induced dissociation of H_2_O molecules occurred only in ice VII among the dense ices, showing a maximum dissociation yield around 14 GPa. The pressure variation of the dissociation yield will be discussed in connection with that of the electric conductivity having a maximum value at the corresponding pressures.

## Methods

Deionized water (Wako Pure Chemical Industries) was used for high pressure experiments with a diamond anvil cell (DAC). Water and ruby chips were placed in a sample chamber, 0.2 mm in diameter and <0.1 mm in thickness, made by drilling a beryllium metal gasket, and compressed between the opposed diamond anvils to a target pressure. Pressures inside the sample chamber were monitored via the shift of the ruby *R*_1_ fluorescence line^10^ and its distribution was found to be less than 10%. An X-ray beam with energy of ~10 keV was used to irradiate the sample through the beryllium gasket at BL12XU in SPring-8^11^. We made five experimental runs up to 43 GPa as summarized in Table 1. The experiments were performed by loading multiple samples to different target pressures and subjecting one sample to irradiation at the target pressure only. Oxygen *K*-edge XRS spectra were measured during irradiation. The incident X-ray energy was scanned from 10.408–10.448 keV, and scattered X-rays with energy of 9.888 keV were detected with an energy loss in the range of 520–560 eV. The incident X-ray energy was calibrated at 9.881 and 11.136 keV with the Ta *L*_2_ and *L*_3_ absorption edges, respectively, and its beams were aligned to pass diametrically through the sample chamber. The analyzer for the scattered X-rays was calibrated with the elastic line at 9.888 keV, which corresponded to the back-scattering energy of a Si 555 spherically bent crystal (Bragg angle *θ*_B_ = 89.32°). The scattering angle was 30°. The incident beam size of ca. 30~60 (H) × 30 (V) μm^2^ (full-width-at-half-maximum, FWHM) was small enough for or comparable to the sample thickness for the low-pressure measurements, but slightly larger than that reduced by compression above 39 GPa. Background signals from the anvils were eliminated efficiently with a clean-up horizontal slit. ORS were measured in 21-GPa and 43-GPa experiments to identify dissociation products after the 10-keV X-ray irradiation. The ORS spectra in the low- (<1700 cm^−1^) and high- (>2500 cm^−1^) frequency regions were measured using a 488.0-nm argon ion laser at 80 mW and a 632.8-nm helium-neon laser at 32 mW, respectively. Angular dispersive XRD profiles were collected with an image plate detector (RIGAKU R-AXIS IV^++^) at BL10XU of SPring-8^12^ with an incident X-ray wavelength of 0.4137 or 0.4134 Å. The incident beam size was ~2 μm (FWHM) for the measurement after irradiation at 21 GPa and ~40 μm for the other measurements.

## Results

### X-ray Raman scattering

**Figure 1:**
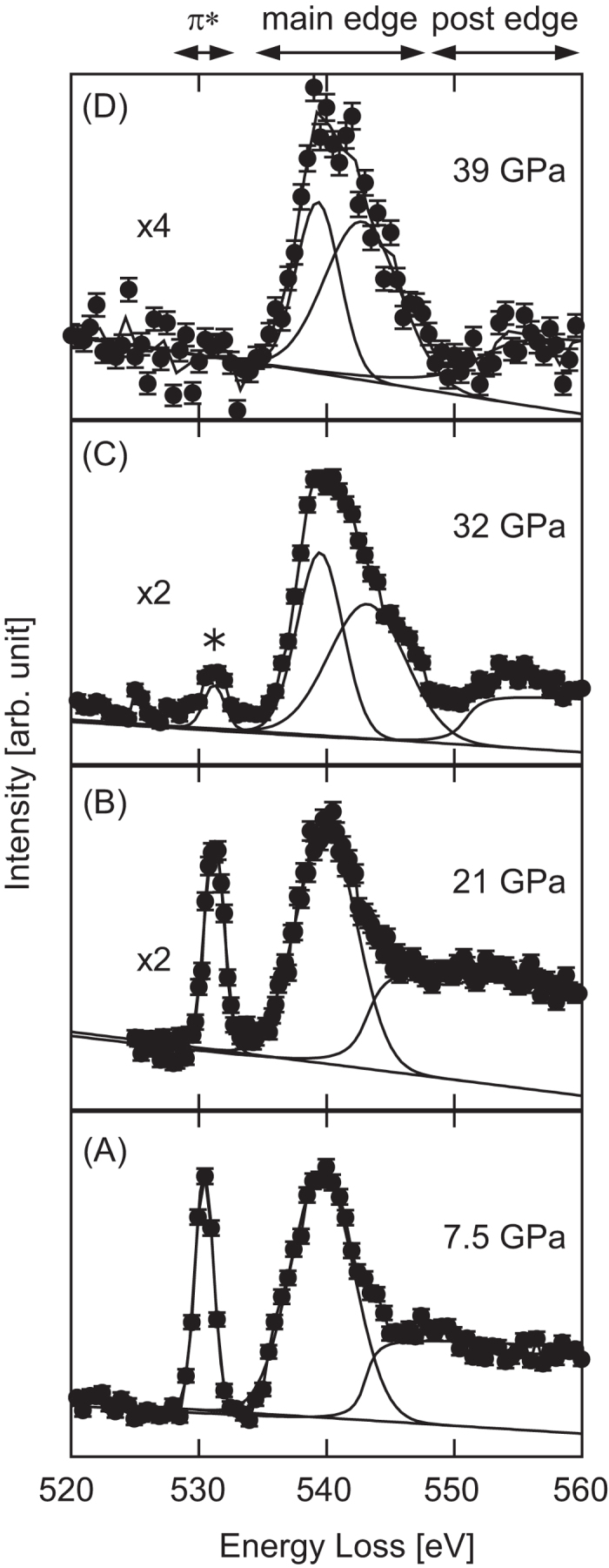
XRS spectra at various pressures. Oxygen *K*-edge XRS spectra of H_2_O at (**A**) 7.5, (**B**) 21, (**C**) 32, and (**D**) 39 GPa, with a typical energy resolution of 1.4 eV. The asterisk shows the location of the signal seemingly derived from oxygen molecules generated by dissociation. Each spectrum were fitted with two or three Gaussian, one arctangent, and one linear base functions shown by solid lines.

Figure 1 shows XRS spectra measured for dense ice up to 39 GPa. Each spectrum was fitted with two or three Gaussian, one arctangent, and one linear base functions. A sharp peak at 530 eV in 7.5-GPa and 21-GPa spectra is assigned to the π* antibonding orbital of the O_2_ molecule, a reaction product of the H_2_O molecular dissociation^8^,^9^. A band located at ~540 eV should consist of two components, the XRS main edge of H_2_O molecule and the peak from the σ* antibonding orbital of the O_2_ molecule^13^. The intensity of the π* peak was as high as that of the band at ~540 eV at these pressure conditions. Since the spectral profiles remained basically unchanged after 2 h of X-ray exposure at these pressures, it was considered that the steady state was achieved in the molecular dissociation reaction in the present measurements. The peak apparently became weak at 32 GPa and disappeared at 39 GPa. The band at around 540 eV showed profile change in association with the disappearance of the 530 eV peak. The 540 eV band was able to be well fitted with one Gaussian, showing a rather symmetric shape at 7.5 and 21 GPa. This is probably owing to the nearly equal contribution from the σ* peak of the O_2_ molecule to the XRS main edge of H_2_O molecule^13^. In contrast, the band requires two Gaussians for reproducing the asymmetric shape at 32 and 39 GPa in a manner similar to that of the main edge of high-density amorphous ice^14^ or ice VII at 2.2 GPa^15^; the σ* component fades out with increasing pressure. These spectral changes indicate suppression of the formation of O_2_ molecules via 10-keV X-ray irradiation at 39 GPa.

### Microphotography

**Figure 2:**
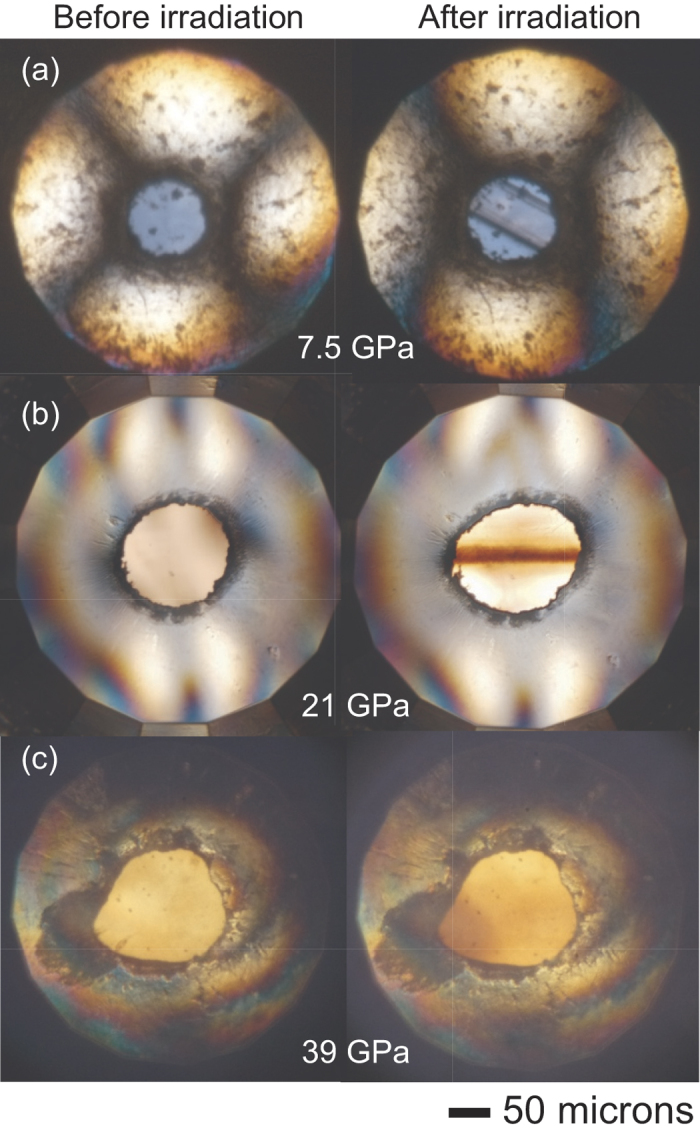
Microphotographs of H_2_O in diamond anvil cells with reflected and transmitted illumination before (left panels) and after (right panels) 10-keV X-ray irradiation at (**a**) 7.5, (**b**) 21, and (**c**) 39 GPa pressures. The X-ray is incident from the upper left in (**a**), the left (**b**), and the lower left in (**c**). The color where the incident X-ray passed changed at 7.5 and 21 GPa whereas there was almost no change observed at 39 GPa. The scale bar is 50 μm.

Figure 2 shows microphotographs of dense ice taken before and after X-ray irradiation. The specimen irradiated at 7.5 GPa shows a slightly damaged portion running from the upper left to the lower right. The width is about 60 μm corresponding to that of the incident X-ray beam. An X-ray irradiated path is definitely seen in a photograph taken at 21 GPa. Ice turns from colorless to dark brown along the path 22 μm in width. Such deterioration of ice by X-ray irradiation has been reported in the previous studies as well^8^,^9^. In contrast, a definite track is not seen in a 39 GPa photograph although slight color change spreads over a wide area of the specimen. Molecular dissociation hardly occurs at 39 GPa. The different color of X-ray path would reflect the high pressure state of O_2_ molecules in the dissociation product. It has been reported that the β-phase of O_2_ exhibits a color of pink at 7.5 GPa and 300 K^16^ and the ε-phase stable above 10 GPa is dark red^16^.

### X-ray diffraction

**Figure 3:**
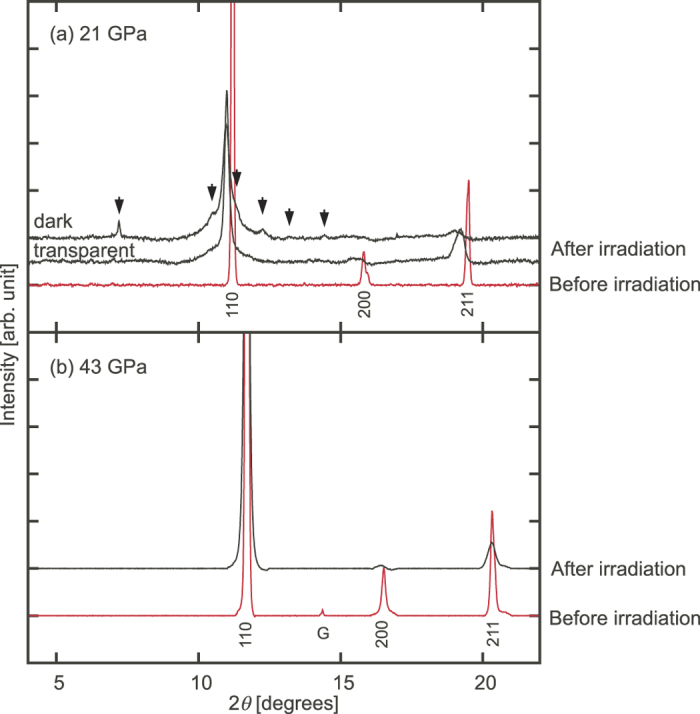
XRD patterns (background subtracted) at (**a**) 21 and (**b**) 43 GPa. The red broken and black solid lines in the XRD patterns indicate before and after irradiation, respectively. Diffraction indices of ice VII with cubic symmetry are shown below the patterns. Numbers above black arrows in (**a**) are diffraction indices of ε-O_2_ (*a* = 7.86(2), *b* = 5.73(2), *c* = 3.58(1) Å, *β* = 113.0(3)°). The letter “G” in (**b**) indicates the diffraction peak of beryllium 101. The incident X-ray energy was 30.0 keV.

XRD patterns taken before and after 10-keV irradiation at 21 and 43 GPa are shown in Fig. 3. Peaks of pre-irradiated ices are indexed with a body-centered-cubic (bcc) lattice, giving lattice constants of 2.998(4) and 2.873(4) Å at 21 and 43 GPa, respectively. These lattice constants correspond to 21.9 and 39.5 GPa, respectively, using the EoS for ice VII in ref. 2. At the irradiated part, the trace of the X-ray path became dark after irradiation at 21 GPa. The dark part approximately 20 μm in width and 150 μm in length was clearly distinguished from the non-irradiated transparent part. The XRD pattern measured for the dark part shows additional peaks at 2*θ* = 7.2° and 12.3° and shoulders at both sides of the 110 peak of ice VII basically in consistent with those reported in the earlier work^8^ and clear evidence of the molecular dissociation. All the peaks can be assigned to the Bragg reflections of bcc ice or ε-O_2_^16^. The present results does not necessarily rule out that the dissociation product is an alloy of H_2_ and O_2_ molecules^8^ because other peaks were not observed for some reason. However, let us just compare the present XRD pattern to ε-O_2_ and ice VII here. The bcc lattice constant was 3.073(7) Å for the darken ice, slightly larger than 3.054(7) Å of the transparent ice. The observed lattice constants *a, b, c*, and *β* of ε-O_2_ were *a* = 7.86(2), *b* = 5.73(2), *c* = 3.58(1) Å, and *β* = 113.0(3)°, whereas those of pure ε-O_2_ at 21 GPa are 7.683, 5.454, 3.650 Å and 116.3°^17^. In addition, the peak widths are significantly broad for the irradiated dark part and even for the non-irradiated transparent part.

The XRD pattern taken at 43 GPa after irradiation is almost identical to that before irradiation, and no additional peaks are observed (Fig. 3b). The X-ray passing path did not turn to dark. The bcc lattice constant was 2.874(3) and 2.880(9) Å for ices before and after irradiation, respectively. The bcc sublattice of oxygen maintained even after irradiation though the peak widths became broader than those before irradiation. This peak broadening may indicate that the lattice of ice was disturbed by O_2_ or H_2_ molecules produced slightly by irradiation even at this condition or that the size of H_2_O crystallites became smaller by the partial dissociation.

### Optical laser Raman spectroscopy

**Figure 4:**
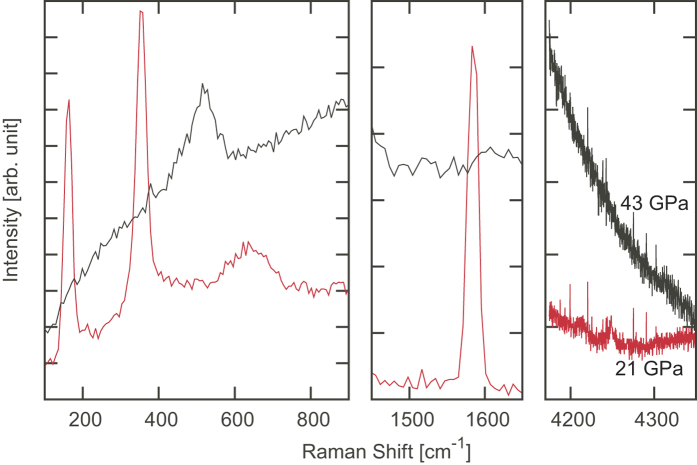
ORS results for H_2_O after 10-keV X-ray irradiation. The red and black spectra were obtained at 21 and 43 GPa, respectively.

The formation of O_2_ molecules in association with the molecular dissociation is confirmed from ORS spectra. Figure 4 shows ORS spectra measured for the central part of the sample after X-ray irradiation at 21 and 43 GPa. In the 21-GPa spectrum, three peaks in the lower frequency region, a sharp intense peak around 1570 cm^−1^, and one weak peak around 4250 cm^−1^are assigned to two O_2_ librons and an overtone (ν_L1_, ν_L2_, and 2ν_L2_), a O_2_ vibron^18^, and a H_2_ vibron^9^,^19^, respectively. The weak H_2_ vibron was probably due to diffusion of H_2_ molecules into the surrounding ice; this is suggested from the peak broadening of an X-ray diffraction pattern observed for the transparent area or the outside area of the X-ray irradiation path (Fig. 3a). In contrast, only one peak related to lattice phonons of ice VII (B_1g_ + E_g_)^20^ is observed at 530 cm^−1^ and either the O_2_ or H_2_ vibron peak is not observed in the 43-GPa spectrum; the dissociation reaction of H_2_O molecules was strongly suppressed.

## Discussion

We attempt to estimate the yield of the dissociated H_2_O molecules from the XRS spectra so as to discuss the nature of the X-ray-induced dissociation. Several assumptions are made with respect to the scattering cross section of the XRS and the reaction formula. Assuming that the initial state comprises only the 1s core electrons of oxygen, the scattering intensity of the oxygen *K*-edge XRS spectrum between 528 and 548 eV can be expressed as


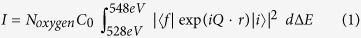


where *N*_*oxygen*_ is the number of oxygen atoms involved in scattering events and C_0_ is a constant depending on the experimental condition. *Q* and *r* are the scattering vector and the electron position operator of the oxygen 1*s* orbital, respectively. The initial and final states of the electronic system are expressed as 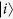 and 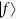, respectively.

We consider that value *I* observed at each pressure is only a sum of those of ice and the dissociation product from the following discussion. Let us define deposited energy as


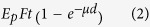


where *E*_*p*_ is the energy of the photon irradiated on the sample (10 keV), F is the flux (typically 1 × 10^13^ photons/s multiplied by the 10-keV X-ray transmission of the beryllium gasket: 0.77), t is exposure time, *μ* is the absorption coefficient of a photon with energy *E*_*p*_, and *d* is the sample diameter (~100 μm: corresponding to the path length of the X-ray). The deposited energy is ~8.2 J after 2 h of X-ray exposure at 7.5 GPa. Assuming this beam is concentrated in a 30 × 30 μm^2^ area, the fluence would be approximately 9.1 × 10^5 ^J/cm^2^. Since the total deposited energy at each pressure is much more than 8.2 J (see Table 1), the XRS spectrum is obtained from the irradiated part of the steady state sample.

The dominant dissociation product obviously contains O_2_ molecule and hence each XRS spectrum can be decomposed into three components: the π* and the σ* peaks of O_2_ and the main edge of H_2_O. Here let us assign *A*, to the area of the π* peak around 530 eV, and *B* and *C* to the areas of the σ* peak and the main edge of H_2_O, respectively, both of which are located between 540 and 548 eV as an overlapped band. Since the scattering intensities only depend on the numbers of oxygen atoms involved in a series of the scattering events, (*A* + *B*)/(*A* + *B* + *C*) should be proportional to *N*_oxygen_product_/(*N*_oxygen_product_ + *N*_oxygen_ice_), where *N*_oxygen_product_ and *N*_oxygen_ice_ are the number of oxygen atoms belonging to the dissociation product and the dense ice, respectively.

**Figure 5:**
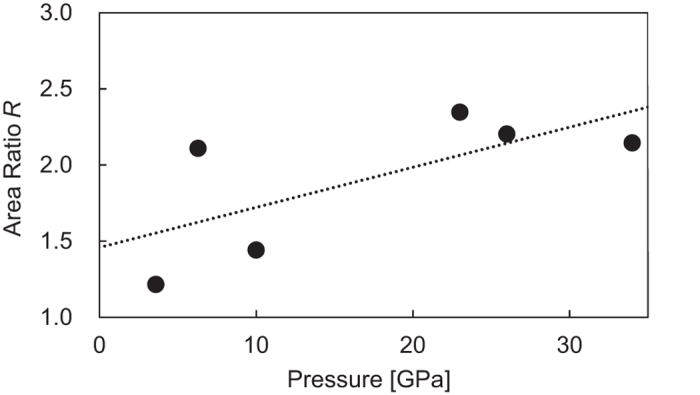
Pressure variation of the ratio of the π* peak area to the σ* band area of pure oxygen from the oxygen *K*-edge XRS spectra^13^. A line, *R* = 1.46 + 0.0263∙*P* [GPa], was fitted to the data.

To estimate *B* and *C* from the observed XRS spectra spectrum, we use the ratio *R* of the σ* band area to the π* peak area for pure oxygen. The pressure variation of the area ratio is approximated as


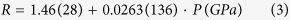


from literature data^13^ (Fig. 5). The numbers in parentheses in Eq. 3 are fitting uncertainty. Note that the value *R* depends on the distribution of the molecular axis of O_2_. Here we assume that the distribution was uniform in both of the previous^13^ and present studies. Accordingly, the ratio of the dissociation product area (*A* + *B*) to the observed area (*A* + *B* + *C*) can be expressed as


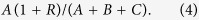


Uncertainty in the area evaluation from XRS spectra was typically 10%. We consider the estimated yields from the present result are saturated values. The yields estimated from the reported XRS spectra^8^ might have been underestimated because the steady state of the dissociation reaction was probably not achieved in their irradiation experiments. Therefore, the estimated dissociation yields calculated in Ref.^8^ are lower bounds.

**Figure 6:**
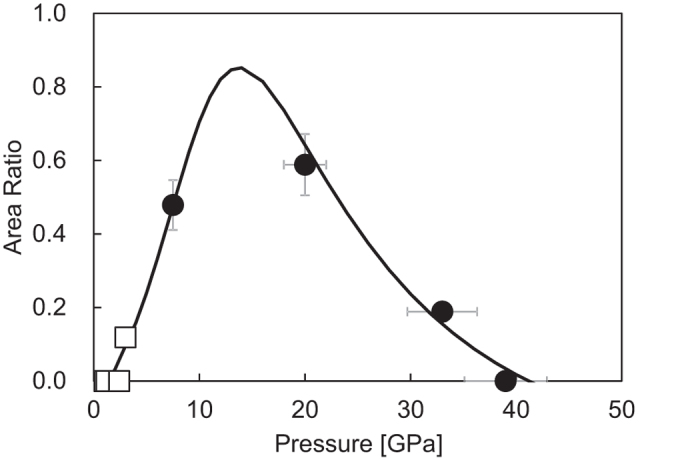
Yields of the dissociation product estimated from the area ratios (*A* + *B*)/(*A* + *B* + *C*). Solid circles and open squares represent data from the present study and ref. 8, respectively. The bold line is the model for the yield using two Arrhenius equations.

The ratio of the dissociation product *(A* + *B*)/(*A* + *B* + *C)* calculated from the present results and the previous results^8^ is plotted as a function of pressure in Fig. 6. Four points from the previous results^8^, each of which indicates the dissociation yield at a target pressure only as well as those obtained by the present experiments, are added in Fig. 6. The dissociation yield rises at about 2 GPa and reaches to a maximum around 14 GPa. With further increase in pressure, the yield decreases gradually and becomes zero at about 40 GPa.

The appearance of the maximum in the dissociation yield would be attributed to “instability” of the hydrogen bond induced in dense ice around 15 GPa, since some *anomalies* in the equation of state and the vibrational frequencies^4^,^5^,^6^, and the maximum electric conductivity were observed in the corresponding pressure region^7^. Among them the electric conductivity with proton motions seems most probable event to be connected with the molecular dissociation. Along this context, we discuss a possible process for the X-ray-induced molecular dissociation.

If the X-ray induced dissociation was related to hydrogen mobility in dense ice, the dissociation yield could show Arrhenius behavior. Although there is no data at different temperatures, we attempted to fit the X-ray induced dissociation yield to the following equation:


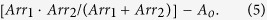


*A*_*o*_ is an offset term so that the model function yields a finite value at a zero limit of the dissociation yield and *Arr*_*i*_ (*i* = 1 or 2) is given by


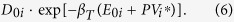


**Figure 7:**
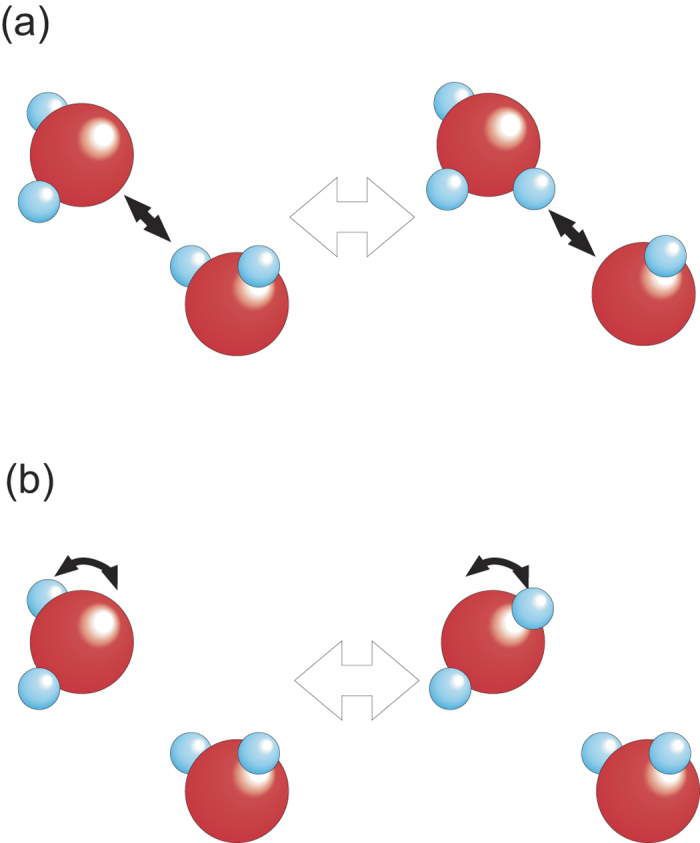
Schematics of two types of hydrogen motion in dense ice. (**a**) Motion between double-well minimums along hydrogen bonding. This is related to the motion of ionic defects. (**b**) Molecular rotation type motion.

*D*_*0i*_*, β*_*T*_*, E*_*0i*_*, P,* and *V*_i_* are the prefactor, thermodynamic beta, activation energy, pressure, and activation volume, respectively. Here we assume a constant value for *V*_i_* over the measured pressure range of 0–40 GPa and describe it in meV/GPa unit. Now, *Arr*_*1*_ and *Arr*_*2*_ can be related to the probability of the two type of hydrogen motion. The first process (*Arr*_*1*_) is hopping between double-well minimums along a hydrogen bond (Fig. 7a), which is related with the mobility of ionic defects in the electrical conductivity^7^. The second (*Arr*_*2*_) is hopping from a site between one O-O nearest pair to another site between another O-O pair (Fig. 7b), which is related with the mobility of rotational defects in the electrical conductivity^7^. *D*_*0i*_ and *E*_*0i*_ cannot be uniquely determined owing to the measurements only at 300 K in the present study and hence two *E*_*0i*_ are fixed to those determined by the electrical conductivity measurements^7^ while two *D*_*0i*_ remain as adjustable parameters.

The pressure variation of the yield is reproduced with this model (Fig. 6). The optimized parameters are listed in Table 2. The model function with the optimized parameters gives a maximum value at 14 GPa. The absolute values of the activation volumes obtained in this study (−6.47 ± 2.59 and 1.78 ± 0.81 meV/GPa) are smaller than those in the electrical conductivity (−36.4 and 25.9 meV/GPa)^7^ by one order of magnitude. Difference in the corresponding activation volumes may indicate that X-ray suppresses the mobility of the ionic defects and enhances that of the rotational defects.

In summary, the present results concluded that the X-ray-induced molecular dissociation observed in dense ice only occurs in ice VII. The dissociation yield can be interpreted in terms of two types of hydrogen mobility in the body-centered oxygen sublattice: the molecular rotation type and the double-well hopping type. The dominant hydrogen behavior changes from the former to the latter at 14 GPa.

## Additional Information

**How to cite this article**: Fukui, H. *et al*. Suppression of X-ray-induced dissociation of H_2_O molecules in dense ice under pressure. *Sci. Rep.* **6**, 26641; doi: 10.1038/srep26641 (2016).

## Figures and Tables

**Table 1:** Summary of experimental conditions.

**Sample #**	**Pressure (GPa)**	**Exposure Time (h)**	**Absorption Coefficient (cm**^**−1**^)	**Deposited Energy**^a^ **(J)**	**Measurement**^b^
1	7.5 ± 0.4	25	9.708	102	XRS
2	32 ± 1.6	8.5	12.82	45.5	XRS
3	39 ± 2.0	13	13.33	72.1	XRS
4	43 ± 2.2	23	13.70	131	XRD, ORS
5	21 ± 1.1	15	11.76	73.9	XRS, XRD, ORS

^a^See text.

^b^XRS: X-ray Raman scattering, XRD: X-ray diffraction, ORS: optical laser Raman spectroscopy.

**Table 2:** Optimized parameters of the yield of the X-ray-induced dissociation of H_2_O molecules in dense ice.

		***Arr***_**1**_	***Arr***_**2**_
*D*_0_	[10^8^]	16.0 ± 19.9	9.81 ± 3.95
*E*_0_	[eV]	0.596^a^	0.503^a^
V*	[meV/GPa]	−6.47 ± 2.59	1.78 ± 0.81

*A*_o_: 0.205 ± 0.219

^a^Fixed to values in ref. 7.
